# Assessing the Radiological Density and Accuracy of Mandible Polymer Anatomical Structures Manufactured Using 3D Printing Technologies

**DOI:** 10.3390/polym12112444

**Published:** 2020-10-22

**Authors:** Paweł Turek, Grzegorz Budzik, Łukasz Przeszłowski

**Affiliations:** Faculty of Mechanical Engineering and Aeronautics, Rzeszów University of Technology, 35-959 Rzeszów, Poland; gbudzik@prz.edu.pl (G.B.); lprzeszl@prz.edu.pl (Ł.P.)

**Keywords:** polymer materials, additive manufacturing, radiological density, accuracy, mandible

## Abstract

Nowadays, 3D printing technologies are among the rapidly developing technologies applied to manufacture even the most geometrically complex models, however no techniques dominate in the area of craniofacial applications. This study included 12 different anatomical structures of the mandible, which were obtained during the process of reconstructing data from the Siemens Somatom Sensation Open 40 system. The manufacturing process used for the 12 structures involved the use of 8 3D printers and 12 different polymer materials. Verification of the accuracy and radiological density was performed with the CT160Xi Benchtop tomography system. The most accurate results were obtained in the case of models manufactured using the following materials: E-Model (Standard Deviation (SD) = 0.145 mm), FullCure 830 (SD = 0.188 mm), VeroClear (SD = 0.128 mm), Digital ABS-Ivory (SD = 0.117 mm), and E-Partial (SD = 0.129 mm). In the case of radiological density, ABS-M30 was similar to spongious bone, PC-10 was similar to the liver, and Polylactic acid (PLA) and Polyethylene terephthalate (PET) were similar to the spleen. Acrylic resin materials were able to imitate the pancreas, kidney, brain, and heart. The presented results constitute valuable guidelines that may improve currently used radiological phantoms and may provide support to surgeons in the process of performing more precise treatments within the mandible area.

## 1. Introduction

Nowadays, 3D printing technology is among the rapidly developing methods applied to manufacture even the most geometrically complex models. Manufacturing a model with 3D printing techniques consists of adding materials layer-by-layer, thus gradually forming the desired shape [1,2]. The input model is designed by means of computer-aided design (CAD) systems [3,4] or reverse engineering (RE) methods [5,6] and is saved in standard triangulate language (STL) format. Before the actual object is manufactured, it is divided into layers. The single-layer thickness largely depends on the additive manufacturing method used. Depending on the manufacturing technology, as well as the dimensions of the object and the complexity of its geometry, manufacturing ready-made models using 3D printing techniques may take several hours or even several days. As a result, this manufacturing technology is mainly used for piece production [7,8,9]. At present, there is a wide variety of ways to manufacture models by applying 3D printing techniques (Table 1). Differences in their functioning occur mainly in the way subsequent layers are cured and the type of material used. Each 3D printer has specific characteristics and requirements related to the working conditions (e.g., material, environmental conditions, printing temperature, and finishing of the model) [1,2,10,11]. Models made with 3D printing techniques are used in the aviation [12,13,14], automotive [15,16], medical [17,18,19], and dental industries [20,21,22], as well as in architecture [23] and agriculture [24].

Materials currently used in the 3D printing process include metals [25,26], polymers [27,28,29], ceramics [30,31], and composites [32,33]. However, polymer materials have also been increasingly used. This is due to the wide availability of polymer materials with different mechanical properties related to biocompatibility with human tissues, among others. Polymers can be divided into thermoplastics and thermosets. Thermoplastics are polymers that can be heated above a certain temperature and which solidify when cooled down. Once solid, they can be remelted and reformed again. Thermoplastics are best suited for the production of ready-made products and test prototypes. Some thermoplastic materials have good mechanical properties, as well as high impact abrasion (e.g., polyetheretherketone (PEEK)) [34,35] and chemical resistance (e.g., polylactic acid (PLA)) [36]. Thermosetting resins cannot be melted and reshaped by heating once they are solidified. When subject to high temperatures they will decompose. Thermosetting resins are better suited for aesthetically critical applications because they can be used to produce smooth-surface parts, such as in injection molding. They generally have high stiffness but are more brittle than thermoplastics, so they are not suitable for functional applications [37,38,39]. The use of polymer materials in the medical industry has grown rapidly in recent years. At present, polymer materials are used to 3D print anatomical structures [40,41,42], surgical templates [43,44], implants [45,46], scaffolds [47,48], and tools for patient rehabilitation [49,50].

Recently, there has also been a significant increase in research on the accuracy of manufactured anatomical structures, including those used in surgical processes, e.g., in the craniofacial area. These studies mainly concern accurate dimensional and geometric analysis [41,51,52]. In the literature, research on changes in linear [51,52] and geometric dimensions [40,41,42] also applies to models made of polymer materials. Verification of characteristic linear dimensions is carried out using a caliper [53,54,55,56], coordinate machine [57,58], and measuring arm [59,60]. In the process of evaluating geometric errors, optical systems are used [41,42,61]. There are also publications that take into account the use of tomographic systems in the process of assessing the accuracy [62] and radiological density of 3D printed phantoms used in medical diagnostics [63,64,65]. In this case, the most frequently analyzed polymer materials are acrylonitrile butadiene styrene (ABS) [64,66,67], polylactic acid (PLA) [66,67,68,69], and acrylic resins [65,70]. 

Even though new types of polymer materials [71,72] are being discovered and there is wide access to various 3D printing techniques [1,2], none of the currently used techniques dominate in the area of craniofacial applications. This mainly is due to the different properties of the polymer materials used in manufacturing diagnostic phantoms [68,69,70], which do not necessarily reflect the radiological density of human tissues and the requirements for accurate manufacturing of the anatomical structure or surgical template [48]. Currently, the literature lacks studies conducted on a wider group of polymer materials that at the same time take into account analyses of the radiological density and accuracy of manufactured anatomical structures. Research in this area will deepen the knowledge on the fields of medicine and technical sciences. As a result, it will be possible to select specific 3D printing techniques in terms of the accuracy of a model, so as to increase the precision of surgical procedures related to the craniofacial area, among others. Additionally, ensuring the assessment of radiological density will allow for future developments of mandible geometry phantoms to consist of elements with densities similar to those of human tissues and the most common cysts and tumors in the mandible area (e.g., inflammatory cysts or ameloblastomas). It is particularly important to initiate research on the development of tomography phantoms in the mandible area to more precisely diagnose tumorous lesions. Delayed diagnosis and detection of a tumor (e.g., place and volume) within the mandible area most often leads to removal of the pathogenic tissue, consisting of resectioning a part of the mandible bone. Reconstruction of the mandible geometry after resection, including on an iliac or fibular bone graft, does not allow for full functionality of the mandible because it is the only moving bone in the craniofacial area subjected to multidirectional dynamic loads during the biting and chewing process [41,43]. Therefore, it is important to analyze the available polymer materials, in terms of the radiological density and accuracy of manufacturing, in order to use these materials in the future and create radiological phantoms of the mandible to test the occurrence of various clinical pathologies in this area.

## 2. Materials and Methods

**Figure 1:**
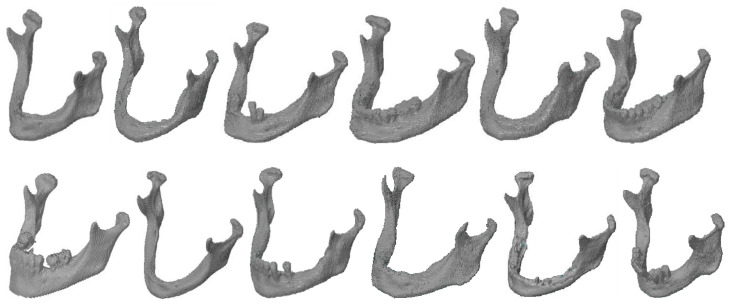
The anatomical structures of the mandible.

The study included 12 different anatomical structures of the mandible, the geometries of which were obtained by reconstructing digital imaging and communications in medicine (DICOM) data from the Siemens Somatom Sensation Open 40 Multi Detector Computed Tomography (MDCT) system scanner installed in the Regional Clinical Hospital No. 1 for all patients at the Frederic Chopin in Rzeszow. The Hospital gives permission to use the DICOM data as a part of the publication. The standard “head routine” protocol for diagnosing the craniofacial area was used in the measurement process. Then, in the course of reconstructing the mandible geometry using ITK-Snap software [72], segmented three-dimensional models were saved in STL format (Figure 1).

**Figure 2:**
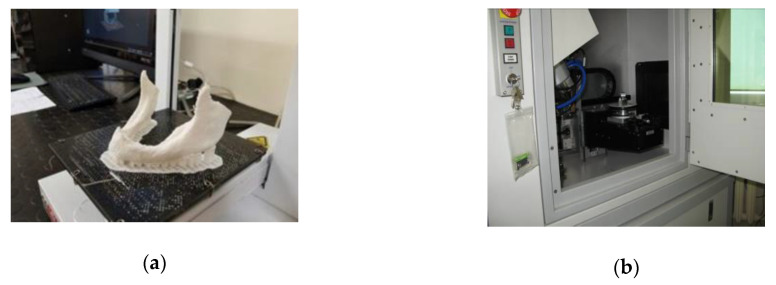
The process of 3D printing and geometry digitalization: (**a**) Prusa MK3s 3D printer; (**b**) CT160Xi Benchtop tomography system.

The process of manufacturing the 12 anatomical mandible structures involved using 12 different polymer materials that are most commonly applied in the process of 3D printing of anatomical structures and elements when constructing radiological phantoms (Table 2). These structures were manufactured at the highest resolution possible for the 3D printers and with the model being fully filled inside. During printing, all models were oriented in the same way in the printer space, as shown in Figure 2a. The goal was to manufacture the side part of the mandible as accurately as possible, as it is the area to which surgical plates are most often bent when planning the procedure.

Geometric accuracy and radiological density were verified with the CT160Xi Benchtop tomography system (Nikon Metrology, Herts, United Kingdom) (Figure 2b). The CT160Xi Benchtop tomography system is equipped with a lamp that generates an X-ray beam. It has a tungsten cathode and anode with an accelerating voltage range of 40–160 kV, an intensity range of 0–500 μA, and a maximum power of 60 W. The image is detected on the detector matrix and consists of 1900 (horizontal) × 1516 (vertical) pixels, which at the linear pixel size of 0.127 mm gives a surface size of 24.2 × 19.3 cm. In addition, the system includes a rotary table that allows for linear and angular movement and positioning of the measuring element. According to the Verein Deutscher Ingenieure (VDI)/Verband der Elektrotechnik (VDE) 2630 specifications, the accuracy of the computer tomography was verified. Computed tomography scans of the mandible were acquired at 80 kVp, 131 μA, and a voxel size 0.05 × 0.05 × 0.05 mm. The model in the tomograph space was oriented in the same way as shown in Figure 2a.

**Figure 3:**
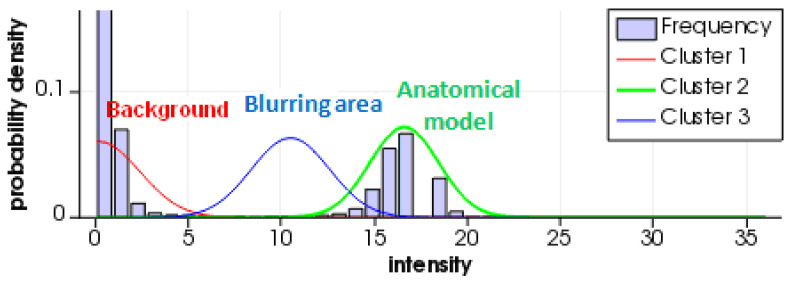
The process of grouping data for the Precimid 1170 material.

The anatomical structures of the mandible were reconstructed using ITK-Snap software. Using the prepared data, segmentation was performed using a Gaussian mixture model-based clustering algorithm [73]. This algorithm assumes that the distribution of each element in a random sample consists of a combination of less complex distributions that can be approximated to a Gaussian curve. Based on the analysis of the histogram showing the image pixel intensity distribution, the matching process was carried out (Figure 3). 

Then, a group of pixels representing the anatomical structure of the mandible was selected and subjected to geometric reconstruction using the active contour algorithm. This algorithm adjusts the contour to the outline of the anatomical structure determined in the segmentation process, allowing us in the end to fully reconstruct the geometry of the mandible.

## 3. Results

**Figure 4:**
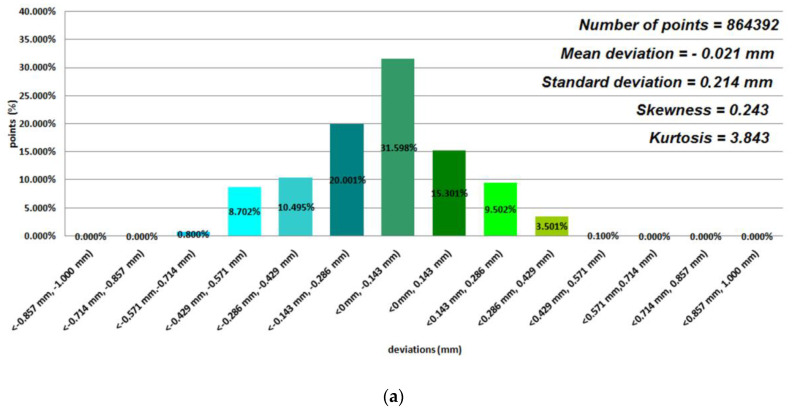

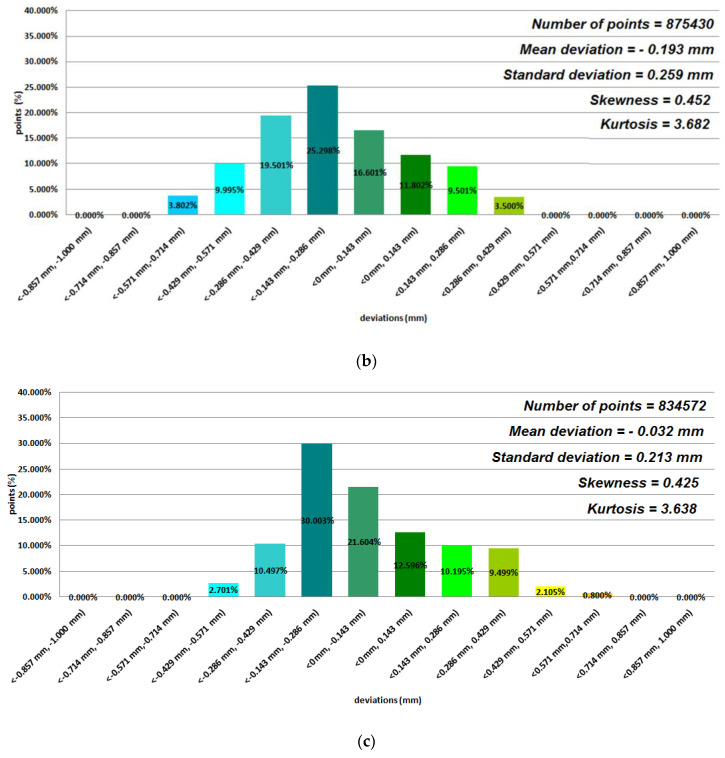
Histograms representing average results of the 12 mandible structures: (**a**) ABS M-30 material; (**b**) PC-10 material; (**c**) Polylactic acid (PLA) material.

**Figure 5:**
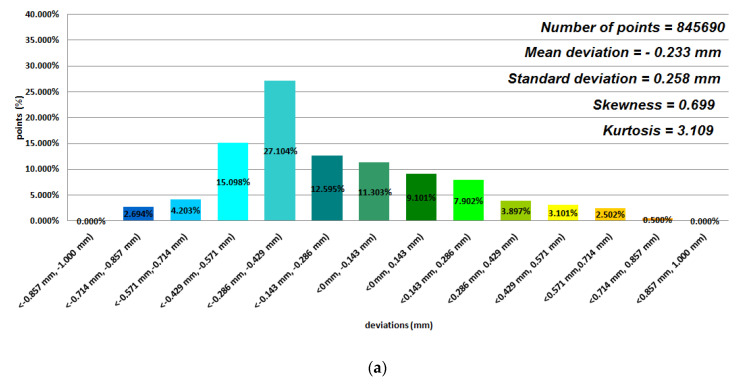

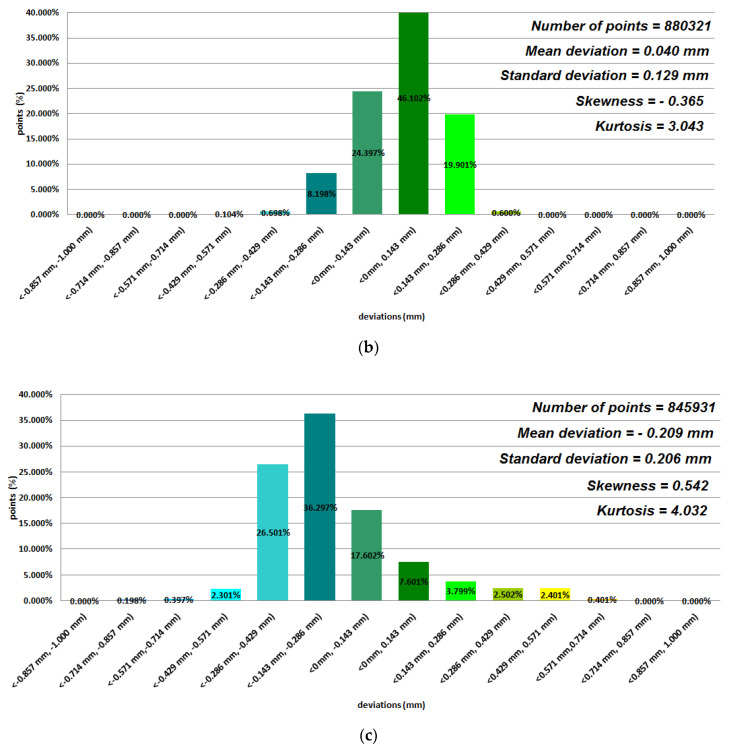
Histograms representing average results of the 12 mandible structures: (**a**) Polyethylene terephthalate (PET) material; (**b**) E-Partial material; (**c**) E-Denstone material.

**Figure 6:**
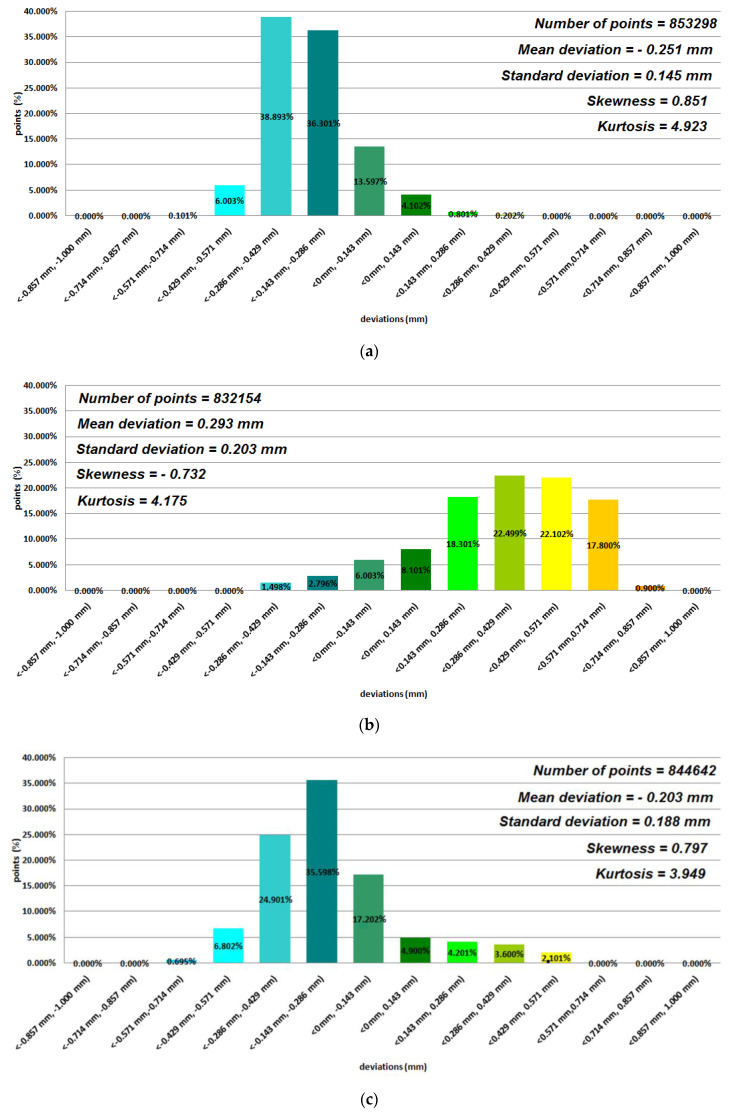
Histograms representing average results of the 12 mandible structures: (**a**) E-Model material; (**b**) Precimid 1170 material; (**c**) FullCure 830 material.

**Figure 7:**
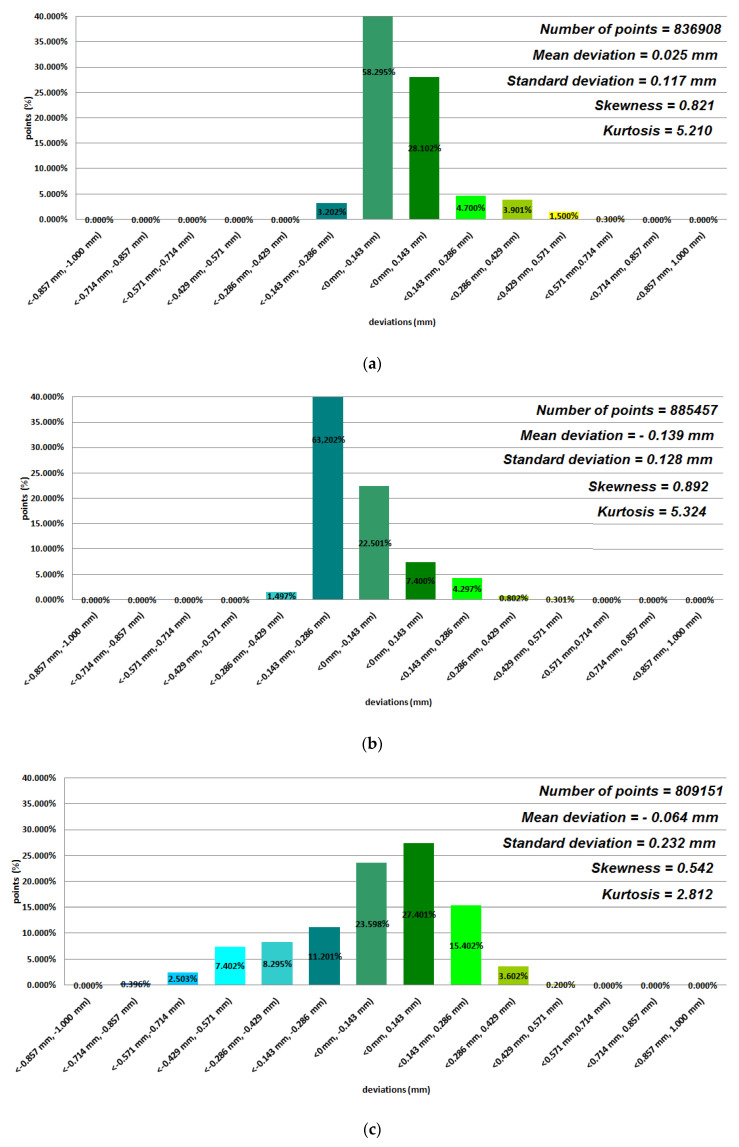
Histograms representing average results of the 12 mandible structures: (**a**) Digital ABS-Ivory material; (**b**) VeroClear material; (**c**) RGD720 material.

Data representing 12 models of anatomical mandible structures were used to assess the manufacturing accuracy with the Focus Inspection software for the 12 selected polymer materials (Figure 4, Figure 5, Figure 6 and Figure 7), while the radiological density was assessed using ITK-Snap software (Table 3). Until now, no studies on such a wide range of polymer materials have been presented in one publication.

Based on the average statistical analysis of the mandible structure accuracies for the selected 12 polymers materials, the mean deviation, standard deviation, skewness, and kurtosis were determined with histograms. The normality of the data was assessed with the Shapiro–Wilk test (Figure 4, Figure 5, Figure 6 and Figure 7) and the value of the statistics test was W = 0.806 (*p* = 0.05 and n = 12). This value was higher than the critical value, and as a result we did not reject the hypothesis of the normal distribution. Accordingly, all obtained distributions were treated as normal. Each of the presented distributions was unimodal. The best results were obtained for models manufactured using the following materials: E-Model (Standard Deviation (SD) = 0.145 mm), FullCure 830 (SD = 0.188 mm), VeroClear (SD = 0.128 mm), Digital ABS-Ivory (SD = 0.117 mm), and E-Partial (SD = 0.129 mm). However, regarding the mean deviation values for the E-Model and FullCure 830 materials, these showed significant differences from the nominal value of −0.251 mm and −0.203 mm, respectively. This may have been influenced by their fastest shrinkage material process compared to other polymer materials. The highest value of the standard deviation was observed in the case of manufacturing models using FDM and MEM techniques (in the case of Polyethylene terephthalate (PET) and PC-10 materials). This may have also been influenced by the layer thickness. The obtained results were characterized with a positive skew. Only in the case of Precimid 1170 and E-Partial material was a negative skew observed (−0.732 and −0.365, respectively). Regarding kurtosis values, it can be seen that the data distributions were mainly leptokurtic. The exception was the model printed using RGD 720 material. It was noticed that when using the same 3D printer and parameters (orientation in the 3D printer space and layer thickness) but a different material (e.g., in the Fortus 360-mc or Objet350 Connex 3 cases), the accuracy of the printed model changed (Figure 4a,b and Figure 7).

Radiological densities were determined for the most characteristic human tissues [64]. By comparing the obtained results in Table 3, some materials can be assigned to specific human tissues. In the case of materials made using FDM and MEM techniques, ABS-M30 imitated radiological densities similar to those of spongious bone, while PC-10 imitated radiological densities similar to the liver. In the case of the PLA and PET material results in Table 3, these were very similar to the spleen. Considering acrylic resin materials, all radiological densities were between 20 HU and 40 HU. This value imitated the pancreas, kidney, brain, and heart. In the case of the Precimid 1170 material, it characterized the muscle.

When comparing the radiographic densities of the most known tumors and cysts for the mandibular area [74], E-Partial, E-Model, and E-Denstone imitated densities similar to those of ameloblastomas, nasopalatine duct cysts, and inflammatory cysts, respectively. RGD 720 and FullCure 830 imitated dentigerous cysts and keratocystic odontogenic tumors, respectively. The central giant cell lesion radiological density was imitated with the PC-10 material. 

## 4. Discussion

**Figure 8:**
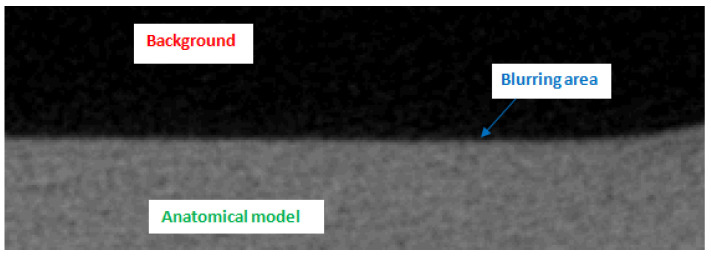
A part of the tomography image with specified areas for the Precimid 1170 material.

In view of the current research being conducted globally, we established that the main factors affecting the quality of the obtained radiological density and accuracy in mapping the geometries of anatomical structures do not solely result from the type of material used in the 3D printing process. In particular, the credibility of the obtained research results was also affected by the digitization stage and the processing of volumetric data [75,76,77]. In the case of carrying out diagnostics using a tomographic system, the measurement parameters (including tube potential, tube current, or pitch) and image reconstruction (including voxel size, convolution kernel, slice thickness) play important roles [75,77,78]. The spatial resolution of the image, which is also related to the voxel dimension, plays a particularly important part in reliably assessing the radiological density and accuracy of a geometric representation [76,78]. As a result of applying data characterized by voxel dimensions measuring 0.05 × 0.05 × 0.05 mm, the partial volume artifact was minimized. As shown in Figure 3, the normal distribution based on the segmentation algorithm that defines the blurring area covers only a small fraction of the pixels representing the image. As a result, it was possible to more accurately indicate the boundary between the background and the pixels defining the anatomical structure of the mandible on the tomographic image (Figure 8). This procedure made it possible not to understate or overestimate the volume of the model geometry subjected to the reconstruction process from tomographic images, and allowed us to estimate the radiological density of the material with greater accuracy.

By analyzing the literature, the radiological density of selected polymer materials can be assessed [64,65,66,67]. However, there have been no studies carried out on such a wide range of polymer materials. Furthermore, 3D printing is becoming increasingly available and offers new opportunities to tailor phantoms for specific clinical and research purposes, including radiotherapy [63,64,65]. It is particularly important to initiate research on developing tomography phantoms of the mandible area to more precisely diagnose tumorous lesions. 

## 5. Conclusions

Currently, 3D printing techniques play important roles in the medical and dental industries. Due to the wide availability of polymer materials used in the 3D printing process, it is possible to manufacture anatomical structures, surgical templates, implants, scaffolds, and tools that support patient rehabilitation. In the present research, the accuracy levels of the mandible anatomical structures and the radiological densities of 12 polymer materials were assessed. The results constitute valuable guidelines that may improve the currently used radiological phantoms and may provide support to surgeons when performing more precise treatments within the craniofacial area. The obtained results are a starting point and further studies should extend the research on the impacts of changing the contrast and spatial resolution of DICOM data on dimensional and geometric accuracy, as well as radiological density. 

## Figures and Tables

**Table 1:** Additive manufacturing processes.

3D Printing Processes	Description	AM Technologies	Application
Vat Polymerization	Selective curing of photo-curable material in a liquid container	Stereolithography (SLA); Digital Light Processing (DLP); Scan, Spin, and Selectively Photocure (3SP); Continuous Digital Light Processing (CDLP)	This technology is most suitable for applications in injection molding [21], jewelry [1], dental [20,21], and medical industries [6], where a smooth surface finish and high accuracy are required
Powder Bed Fusion	Fusing of powder in a bed by melting the selected region	Multi Jet Fusion (MJF), Selective Laser Sintering (SLS), Direct Metal Laser Sintering (DMLS)/Selective Laser Melting (SLM), Electron Beam Melting (EBM)	Powder bed fusion builds functional prototypes with good mechanical properties and is used in aerospace [12,13], automotive [7,25], and medical industries [17,25]
Material Extrusion	Layer-by-layer deposition of molten material	Fused Deposition Modeling (FDM)/Fused Filament Fabrication (FFF)/Melted Extruded Modeling (MEM), 3D Bioprinting	Material extrusion has dimensional accuracy limitations, so it is mainly used in low-cost prototyping [17,19,22]. Industrial systems can also produce functional prototypes from engineering materials [2,28].3D bioprinting focuses on building scaffolds [6,18]
Directed Energy Deposition	Direct fusion of the material	Laser Engineering Net Shape (LENS), Electron Beam Additive Manufacturing (EBAM)	Directed energy deposition technology can be used for repairing or adding material to existing components. This technology is most suitable for applications in aerospace [12,13], automotive [15,16], and medical industries [6,25]
Sheet Lamination	Bonding of individual sheets of material	Laminated Object Manufactured (LOM)	Sheet lamination technology can be used only in ergonomic manufacturing studies [1,7], for visualizing topography [2,3], or for creating architecture models [23] with paper-made objects
Material Jetting	Material deposition and subsequent curing	Material Jetting (MJ), Nanoparticle Jetting (NPJ), Drop On Demand (DOD)	Material jetting is used in lost wax casting and investment casting applications [14], as well as dental [22] and medical industries [6], because it has high accuracy and gives a smooth surface finish
Binder Jetting	Selective dispensing of binder for joining powder in a bed	Binder Jetting (BJ)	Ceramic-based binder jetting can be used typically for manufacturing visual or light-duty functional prototypes (e.g., architectural models) [23]. This technology is not intended for functional applications [1,3,7]

**Table 2:** Selected polymer materials.

AM Processes	AM Technology	3D Printer	Commercial Material Name	Generic Name	Status of Material
Material Extrusion	Fused Deposition Modeling (FDM)	Fortus 360-mc	ABS-M30	Acrylonitrile Butadiene Styrene	Solid-Based
			PC-10	Polycarbonate	
	Fused Filament Fabrication (FFF)	Prusa MK3s	PLA	Polylactic acid	
			PET	Polyethylene terephthalate	
Vat Polymerization	Digital Light Processing (DLP)	Perfactory Vida	E-Partial	Acrylic	Liquid-Based
	Scan, Spin, and Selectively Photocure (3SP)	3Dent–3SP	E-Denstone	Acrylic	
		Xtreme 3SP	E-Model	Acrylic	
Powder Bed Fusion	Selective Laser Sintering (SLS)	TMP Elite 3600	Precimid 1170	Polyamide 11	Powder-Based
Material Jetting	Material Jetting (MJ)	Eden 260V	FullCure 830	Acrylic	Liquid-Based
		Objet350 Connex 3	Digital ABS-Ivory	Acrylic	
			VeroClear	Acrylic	
			RGD720	Acrylic	

**Table 3:** Statistical parameters representing average Hounsfield (HU) values of 12 mandible models.

Polymer Material	Mean Deviation (HU)	Standard Deviation (SD) (HU)
ABS-M30	98.041	5.481
PC-10	57.287	5.576
PLA	48.662	2.995
PET	47.406	8.547
E-Partial	30.126	8.279
E-Denstone	28.594	9.748
E-Model	28.759	10.610
Precimid 1170	16.091	4.348
FullCure 830	29.409	4.775
Digital ABS-Ivory	30.430	3.814
VeroClear	29.055	3.206
RGD720	28.860	6.525
